# The effects of integrating the Manchester Pain Management Model and empowerment education on postoperative rehabilitation after cesarean section: A comprehensive intervention study

**DOI:** 10.1371/journal.pone.0324292

**Published:** 2025-06-16

**Authors:** Hailing Jiang, Ronghua Xu, Lingfang Li, Haijuan Zhu

**Affiliations:** 1 Department of Obstetrics, Hefei Maternal and Child Health Hospital, Hefei, Anhui Province, China; 2 Department of Anesthesia, Hefei Maternal and Child Health Hospital, Hefei, Anhui Province, China; Fundacao Oswaldo Cruz Instituto Rene Rachou, BRAZIL

## Abstract

**Purpose:**

To evaluate the effect of combining the Manchester Pain Management Model (MPMM) with the enabling education model as a comprehensive intervention in maternal rehabilitation after cesarean section.

**Methods:**

A total of 120 women who underwent cesarean section in our hospital from June 2021 to June 2023 were randomly divided into observation group and control group, with 60 cases in each group. The control group received standard nursing measures, while the observation group received comprehensive nursing measures combined with Manchester pain management and enabling education model. A rigorous randomized controlled trial design was employed. Data were analyzed using independent t-tests for continuous variables and chi-square tests for categorical variables, with a significance level set at P < 0.05. Postoperative pain degree (VAS score), postoperative recovery indicators, and nursing satisfaction scores were compared between the two groups.

**Results:**

The pain scores at 4 hours, 12 hours and 24 hours after surgery, the first independent time out of bed activity, the first time to exhaust gas and the length of hospital stay in the observation group were significantly lower than those in the control group (*P* < 0.05), and the success rate of the first breastfeeding and nursing satisfaction were significantly higher than those in the control group (*P* < 0.01).

**Conclusion:**

The combined intervention of the Manchester pain management model and empowerment education significantly improved postoperative pain control, accelerated physiological recovery, and increased overall satisfaction with nursing care. This model may provide an effective new method for rehabilitation management after clinical cesarean section.

## 1. Introduction

Cesarean section (CS) remains a prevalent and often necessary mode of delivery in modern obstetrics, with rates continuing to rise globally. While it can mitigate certain risks associated with childbirth, CS also presents unique postoperative challenges, particularly in pain management and recovery [1]. The pain resulting from CS not only causes significant physical discomfort but can also negatively impact the mother’s psychological state, daily activities, and the establishment of the parent-child relationship, especially in the crucial early stages of breastfeeding and personal care [2].

Effective postoperative pain management and recovery strategies for women undergoing CS are critical aspects of obstetric care. Traditional approaches to pain management often rely heavily on pharmacological interventions, which can be limited by potential side effects and concerns about impacts on infants [3,4]. Moreover, medication alone often fails to address the multifaceted nature of pain, including emotional, cognitive, and sociocultural factors. This gap in comprehensive care highlights the necessity for more integrated approaches to pain management in post-CS recovery [5].

Recent advancements in pain management strategies have led to the development of more holistic models. Among these, the Manchester Model for Pain Management (MPMM) has gained attention for its systematic framework that considers the complex needs of post-surgical mothers. This model proposes a woman-centered approach to pain assessment and management, aligning with the growing emphasis on patient-centered care in medical practice [6]. Complementing pain management strategies, the empowerment education model has emerged as a valuable approach in postoperative care. This model emphasizes improving maternal self-efficacy through education and emotional support, which is crucial for the subjective experience and management of pain [7].

Early educational interventions can enhance women’s understanding of pain, encouraging active participation in their pain management and improving self-care abilities. This shift towards patient empowerment represents a significant departure from traditional, passive patient roles in medical care [8]. Despite the potential benefits of combining the MPMM and empowerment education in post-CS rehabilitation, there is a paucity of research examining the clinical application and effectiveness of this comprehensive intervention approach. The existing literature lacks robust evidence on the actual impact of such integrated programs on post-CS recovery outcomes.

The rationale for focusing on this topic stems from several key factors. The high prevalence of CS and its associated recovery challenges necessitate improved postoperative care strategies. The limitations of traditional, medication-focused pain management approaches highlight the need for more comprehensive, patient-centered interventions. Furthermore, the potential synergistic effects of combining structured pain management (MPMM) with patient empowerment strategies have not been adequately explored in the context of post-CS care. There is also a growing emphasis in healthcare on holistic, patient-centered approaches that address both physical and psychosocial aspects of recovery [9].

This study aims to address this gap by designing, implementing, and rigorously evaluating a comprehensive intervention program that combines the MPMM with empowerment education. We hypothesize that the integration of the MPMM with empowerment education will lead to improved pain management, faster physical recovery, and higher patient satisfaction compared to standard care practices. By examining the effects of this comprehensive intervention on post-CS pain experiences, physical rehabilitation indicators, and maternal satisfaction, this research aims to provide evidence-based guidance for enhancing the postoperative rehabilitation process for CS patients.

The findings of this study have the potential to significantly impact clinical practice by offering a new perspective and approach to post-CS care. If successful, this integrated model could pave the way for more effective, patient-centered rehabilitation protocols in obstetric care, ultimately improving the overall experience and outcomes for women undergoing cesarean sections. This research contributes to the broader goal of optimizing maternal health outcomes and experiences in the critical postpartum period.

## 2. Materials and methods

### 2.1. General information

This study obtained ethical clearance from Hefei Maternal & Child Health Hospital (Approval YYLL2021-YJ009-02-01). All mothers who participated in the study were explained in detail and signed informed consent after fully understanding the purpose and process of the study. Any adverse reactions of the mothers during the study were recorded and dealt with by the requirements of the hospital ethics committee. In this study, 120 parturients who underwent cesarean section in our hospital from June 2021 to June 2023 were selected as research objects. According to random number table method, all parturients were divided into observation group and control group, with 60 cases in each group. All maternity information is strictly confidential and is presented in coded form during data collection and collation to ensure that the privacy of the mother is not violated. Maternal age ranges from 18 to 45 years, gender is female, gestational weeks between 37 and 42 weeks, body mass index (BMI) between 18.5 and 30 kg/m².

Inclusion criteria: (1) at least 18–45 years old; (2) full pregnancy between 37 and 42 weeks of gestation; (3) primiparous or scarred uterine parturients who meet the diagnostic criteria for cesarean section and undergo cesarean section surgery; (4) preoperative and postoperative autonomous language and behavioral ability barrier-free, able to communicate and communicate normally; (5) the mothers and their families fully understand the research content and sign informed consent, agreeing to participate in the study.

Exclusion criteria: (1) serious heart disease, abnormal liver and kidney function, coagulation dysfunction or other major diseases; (2) preoperative chronic pain disease or long-term use of analgesic drugs; (3) women with a history of mental illness or receiving psychiatric treatment; (4) have a history of allergies and allergic reactions to the drugs that may be used in the study; (5) women who were converted to emergency cesarean section during the operation; (6) women who do not want to participate in the study or quit midway.

### 2.2. Research methods

This study employed a randomized controlled trial (RCT) design to evaluate the effectiveness of a comprehensive intervention combining the Manchester Pain Management Model (MPMM) with the enabling education model for maternal rehabilitation after cesarean section. The primary dependent variables in this study were postoperative pain scores (measured using the Visual Analogue Scale), postoperative recovery indicators (including time to first independent out-of-bed activity, time to first flatus, and length of hospital stay), and nursing satisfaction scores.

To ensure the rigor of the study, several measures were implemented. Randomization was conducted using a computer-generated random number table to minimize selection bias. The researchers responsible for data collection and analysis were blinded to group allocation to reduce potential bias. Standardized protocols were developed for both the intervention and control groups to ensure consistency in care delivery.

The control group of 60 women received standard post-operative care procedures, including medical staff referral, routine health education, preoperative consultation and evaluation, and post-operative pain monitoring and management based on maternal needs.

On the basis of standard nursing, 60 pregnant women in the observation group were given enhanced nursing measures combined with MPMM and enabling education mode. The implementation steps are as follows:

(1) Establish an interdisciplinary pain management team. A pain management team consisting of researchers, obstetricians, nursing specialists, anesthesiologists, and psychologists was formed. Its responsibilities included designing care plans to link up maternal pain management, conducting comprehensive and systematic training of nursing staff on qualitative research methods and evaluation of implementation interventions, and collecting and analyzing maternal satisfaction questionnaires.
(2) Construct the interview outline. Guided by MPMM and combined with enabling education, the team constructed the initial interview outline on the basis of existing literature. The outline was optimized according to the preliminary interview data obtained and the opinions of the senior seniority and senior professional title expert group of the hospital. Finally, the following interview questions were identified to explore the post-operative pain experience of women who delivered cesarean section: A: Please review and describe your post-operative pain level and its impact. B: How would you evaluate the existing pain relief measures? What do you usually do when pain occurs? C: How does postoperative pain affect your personal life? D: What recommendations or requirements do you have for postoperative pain management?
(3) Collect interview data. Prior to enrollment, the environment was acclimated and a trusting relationship was established with the mothers to ensure the effectiveness of support and coordination. The potential risks and benefits of the study were explained to the women, and it was made clear that participation in the study would not affect medical treatment and that the interview content was not related to the caregiver’s job assessment. For personal information, anonymous numbers are adopted to ensure that maternal privacy is strictly protected. One-to-one in-depth interviews are conducted in a quiet space within the ward, beginning with the admission of the mother. Adjust the order of inquiry according to the actual situation to ensure the natural flow of the interview. The research focuses on pain management, seeking to capture the real experience and care needs of mothers, and avoid influencing their responses through guided questions. Detailed records of maternal social life background, pain expectations and coping strategies were recorded, and the interview time was controlled between 25 and 30 minutes.
(4) Implementing pain management programs. In order to improve the pain cognition and self-management ability of parturient women, the psychological state and expected goals of parturient women were fully considered, including physical operation demonstration, individualized video teaching and knowledge manual guidance. Help women better participate in pain management through customized goals and plans, including the following areas:
  A. Establish emotional support: Use open inquiry to listen to the worries and expectations of the pregnant women about the preoperative pain, and provide positive incentives and feedback. Mobilize family members to leave messages of support in the “spiritual encouragement wall” to provide maternal spiritual support. Invite experienced women with good pain control to exchange experiences with new women and promote confidence.
  B. Set periodic goals: The mother and her family jointly set short – and medium-term goals for pain relief, such as ensuring adequate rest and carrying out early postoperative activities. Adjust the target according to the actual situation of mothers, so as not to undermine the confidence of mothers.
  C. Develop individualized programs: provide mothers and their families with a clear understanding of the causes, characteristics and possible negative effects of pain through interactive questioning, and provide pain education manuals. This paper introduces the use of visual analogue scale (VAS) to educate parturients to accurately express their pain feelings and effects. Physical simulations and demonstrations introduce self-controlled analgesic manipulation, using cognitive-behavioral techniques such as mindfulness meditation, musical relaxation, and attention-shifting techniques to improve their ability to self-manage pain.
  D. Use the visual analog scale to assess pain at a specified time (e.g., 4, 12, and 24 hours after surgery), and adjust analgesia regimen based on maternal pain feedback and score results, including the appropriate combination of drug and non-drug therapies. For individuals with different response levels, individualized adjustments should be paid attention to in the implementation of the program to achieve the best results (such as timely reminding doctors and adjusting treatment strategies for mothers with high scores or sleep disturbance), and continuous attention should be paid to the overall recovery of the mothers until they are discharged.

### 2.3. Observation indicators

In this study, the degree of postoperative pain, postoperative recovery and nursing satisfaction of parturients in the observation group and the control group were regularly observed and evaluated. The Visual Analogue Scale (VAS) was used to evaluate the pain degree at 4 hours, 12 hours and 24 hours after obstetrics and gynecology, respectively, in the resting and active states. 0 points indicated no pain, 10 points indicated unbearable pain, and the greater the score, the stronger the pain degree. All the women were recorded for the first time, the first independent time out of bed activity, the first time to exhaust gas, the number and proportion of the first successful breast feeding, the postoperative urine volume and the length of hospital stay to evaluate the recovery after obstetrics and gynecology. The Houston Pain Questionnaire (HPOI) was used to assess maternal satisfaction with nursing care. The questionnaire was divided into three parts: pain control method satisfaction, pain education satisfaction and overall nursing satisfaction. The score was 70 points, 50 points and 10 points respectively. The higher the score was, the higher the nursing satisfaction was.

### 2.4. Statistical analysis

Statistical analysis of all data was performed using SPSS 25.0. Mean ± standard deviation was used to describe the measurement data, and independent sample *t*-test was used for inter-group comparison. Counting data were compared between groups using Chi-square tests. In all analyses, *P* < 0.05 was considered statistically significant.

## 3. Results

### 3.1. Comparison of baseline data between the two groups

Table 1 shows the comparison of basic data between the observation group and the control group. There were no significant differences in mean age (*t* = −0.959, *P* = 0.340), BMI (*t* = −0.791, *P* = 0.430) and gestational age (*t* = 0.435, *P* = 0.664) between the two groups. In addition, the proportion of primipara and repeat parturients was 55% and 45% in the observation group and 58% and 42% in the control group, respectively, and there was no statistical difference between the two groups (primipara, ^2^ = −0.316, *P* = 0.712; Multiple parturients, ^2^ = 0.316, *P* = 0.712), indicating that the basic information of parturients and the analysis of follow-up intervention effect were comparable between the two groups.

**Table 1:** Comparison of baseline data between the two groups ($\bar{x}$ ± s, %).

	Age (years)	BMI (kg/m²)	Gestational age (weeks)	Primipara [n(%)]	Polypara [n(%)]
Observation group (n = 60)	30.75 ± 4.35	26.10 ± 3.15	38.72 ± 1.44	33(55%)	27(45%)
Control group (n = 60)	31.50 ± 4.22	26.55 ± 3.08	38.60 ± 1.50	35(58%)	25(42%)
*t*/^2^	−0.959	−0.791	0.435	−0.316	0.316
*P*	0.340	0.430	0.664	0.712	0.712

### 3.2. Comparison of postoperative pain score (VAS) between the two groups

The comparison of visual analogue scores (VAS) between the two groups at resting and active states at 3 time points after surgery is shown in Table 2. The VAS scores of the observation group were significantly lower than those of the control group at 3 time points after surgery, and the VAS scores of the observation group at 4 hours after surgery were 4.05 ± 1.25 and 5.55 ± 1.30 (*t* = −6.443, *P* < 0.001). At 4 hours after operation, the activity was 5.20 ± 1.40 in the observation group and 6.80 ± 1.50 in the control group (*t* = −6.040, *P* < 0.001). A*t* rest and activity 12 hours and 24 hours after surgery, VAS scores in the observation group were also lower than those in the control group, and the difference was statistically significant (*P* < 0.001). These results suggest that a comprehensive intervention combining the Manchester pain management model and enabling education has a significant effect on the relief of postoperative pain.

**Table 2:** Comparison of postoperative pain scores (VAS) between the two groups (mean ± SD).

Time point	State	Observation group (n = 60)	Control group (n = 60)	t	P
4h post-op	Rest	4.05 ± 1.25	5.55 ± 1.30	−6.443	<0.001
	Active	5.20 ± 1.40	6.80 ± 1.50	−6.040	<0.001
12h post-op	Rest	3.15 ± 1.10	4.40 ± 1.20	−5.912	<0.001
	Active	4.30 ± 1.25	5.65 ± 1.35	−5.663	<0.001
24h post-op	Rest	2.25 ± 0.95	3.35 ± 1.05	−5.942	<0.001
	Active	3.40 ± 1.10	4.50 ± 1.20	−5.250	<0.001

### 3.3. Comparison of postoperative recovery indexes between the two groups

The comparison of key indicators of post-obstetric recovery between the observation group and the control group is shown in Table 3. The mean time of the first independent out of bed activity in the observation group was shorter than that in the control group, 22.35 ± 2.60 hours and 24.80 ± 2.75 hours, respectively (*t* = −5.015, *P* < 0.001). The first exhaust time reflected the recovery of intestinal function, and the observation group was 23.40 ± 5.80 hours and 27.85 ± 6.10 hours earlier than the control group, respectively (*t* = −4.095, *P* < 0.001). There was no significan*t* difference in postoperative urine volume between the two groups (*t* = 1.106, *P* = 0.271), indicating *t*hat the combined intervention did not affect postoperative urine volume. The duration of hospitalization in the observation group was significantly less than that in the control group, 4.65 ± 0.88 days and 5.10 ± 1.02 days, respectively (*t* = −2.587, *P* = 0.011), indicating *t*hat the overall recovery was faster in the observation group. In addition, the number and proportion of successful first breastfeeding in the observation group were higher than those in the control group (^2^ = 8.352, *P* = 0.004).

**Table 3:** Comparison of postoperative recovery indexes between the two groups ($\bar{x}$ ± s, %).

	First independent time out of bed activity (hours)	First exhaust time (hours)	Postoperative urine volume (mL)	Hospitalization duration (days)	First successful breastfeeding [n (%)]
Observation group (n = 60)	22.35 ± 2.60	23.40 ± 5.80	2050.50 ± 350.20	4.65 ± 0.88	51(85.0%)
Control group (n = 60)	24.80 ± 2.75	27.85 ± 6.10	1980.25 ± 345.50	5.10 ± 1.02	37(61.7%)
*t*/^2^	−5.015	−4.095	1.106	−2.587	8.352
*P*	<0.001	<0.001	0.271	0.011	0.004

### 3.4. Comparison of results of questionnaire on maternal nursing satisfaction between the two groups

Table 4 shows the comparison between the observation group and the control group in terms of maternal satisfaction with care. The results showed that the average scores of satisfaction with pain control methods in the observation group were significantly higher than those in the control group, 51.63 ± 8.32 and 43.78 ± 5.66, respectively, and the difference was statistically significant (*t* = 6.043, *P* < 0.001). In terms of pain education, the satisfaction of the observation group (38.65 ± 7.52) was significantly higher than that of the control group (33.52 ± 4.85) (*t* = 4.441, *P* < 0.001). In addi*t*ion, the overall nursing satisfaction score of 8.84 ± 1.25 in the observation group was significantly higher than 8.15 ± 0.97 in the control group (*t* = −3.365, *P* = 0.001).

**Table 4:** Comparison of maternal nursing satisfaction scores between the two groups (mean ± SD).

Satisfaction dimension	Observation group (n = 60)	Control group (n = 60)	t	P
Pain control methods	51.63 ± 8.32	43.78 ± 5.66	6.043	<0.001
Pain education	38.65 ± 7.52	33.52 ± 4.85	4.441	<0.001
Overall nursing	8.84 ± 1.25	8.15 ± 0.97	−3.365	0.001

## 4. Discussion

The incision in a cesarean section causes mechanical damage to the wound and surrounding tissue. This damage triggers the release of pain substances caused by inflammation such as bradykinin, serotonin and prostaglandin, which superimposes the stimulation of uterine contraction on the abdominal wall, making the pain more intense [10]. Therefore, for the women who receive cesarean section, the nursing management of postoperative pain is particularly important. Pain is a comprehensive reflection of multiple factors including physiological, social, psychological and cultural factors, so its assessment should be multifaceted to provide a solid basis for matching care measures [11].

It is crucial to consider the cultural context and differences among Chinese women when discussing post-cesarean section care and pain management. In China, traditional beliefs and practices surrounding childbirth and the postpartum period, can significantly influence women’s attitudes towards pain and recovery. These cultural norms may affect women’s willingness to engage in early mobilization or their expectations regarding pain management. Additionally, the level of previous pain management knowledge among Chinese women may vary widely, depending on factors such as education level, urban or rural background, and access to prenatal education.

The specific context of maternal care in China, including the high cesarean section rate and the evolving healthcare system, also plays a role in shaping women’s experiences and expectations. In recent years, there has been a push towards more natural childbirth and reducing unnecessary cesarean sections in China, which may influence how women perceive and prepare for cesarean deliveries when they are medically necessary. Furthermore, the implementation of the two-child and three-child policies has led to an increase in repeat cesarean sections, presenting unique challenges in pain management and recovery for multiparous women.

The MPMM pain management model, when adapted to the Chinese context, offers a comprehensive approach that considers these cultural factors. This model establishes a four-stage systematic management model based on the multifaceted nature of pain perception and experience. In this study, we sought to gain an in-depth understanding of the lifestyle and socio-cultural background of Chinese women undergoing cesarean section, adhering to a maternal-centered principle to provide targeted pain management guidance according to the needs of different women. This approach reflects a professional, systematic, and targeted pain care management strategy that is culturally sensitive and context-specific [12].

Therefore, this study combined the application of enabling education, on the one hand, to provide emotional support for family members and peers, give maternal heart warmth and strength, and promote them to change from passive recipients to active participants [13]. On the other hand, it helps women master effective analgesic methods, and enhances their confidence in coping with pain and self-coping ability. Studies [14] have shown that empowerment education can improve the self-management ability of women who delivered cesarean section and effectively relieve postoperative pain, which is consistent with the findings of this study. Obviously, the combination of MPMM pain care management and empowerment education not only makes the pain management structure more scientific and reasonable, but also more in line with the feelings and needs of women in cesarean section, which is of great significance for achieving more specialized postoperative pain management and reducing the pain feelings of women in cesarean section. As a special group of surgical caesarean section, in addition to relieving postoperative pain, there are multidimensional needs such as early getting out of bed, breastfeeding and so on.

Studies have found that most women consider pain to be a major concern when getting out of bed early, and there is often a lack of education about it [15]. At the same time, Li et al. ‘s study [10] also pointed out that due to factors such as wound pain, postural restriction, lack of awareness of breastfeeding and lack of support, women who gave birth by cesarean section were prone to failure in postpartum breastfeeding. Therefore, pain relief and education promotion are the key factors affecting postoperative recovery. Therefore, this study adopts MPMM pain nursing management and empowerment education for intervention. The former helps women improve their pain self-management ability at the intervention stage, and effectively alleviates postoperative pain through the combination of drugs and non-drugs, thus eliminating negative factors in the recovery process. The latter enriched the content of health education, helped mothers establish scientific concepts of early get-out activity and breastfeeding, enhanced their willingness, confidence and initiative, effectively shortened the time of first independent out of bed activity, and increased the success rate of first breastfeeding [16]. A study discussed the benefits of enhanced recovery protocols after cesarean delivery, noting that multimodal pain control approaches not only reduced opioid consumption but also facilitated early mobilization and improved maternal-infant bonding [17].

With the development of society, medical personnel not only need to have solid medical knowledge, but also should have good communication and humanistic care ability. In this study, the MPMM pain nursing management model and the enabling education model were combined to strengthen the communication between nurses and cesarean women, help to reach emotional resonance and cognitive agreement, and significantly enhance the sense of trust of both parties. Compared with conventional nursing, this method pays more attention to maternal decision-making rights and personality characteristics, overcomes the shortcomings of traditional nursing methods, which emphasize medical care and neglect patients, helps to establish a harmonious nurse-patient relationship, and further improves maternal satisfaction [18,19]. A study on maternal perceptions of cesarean birth care revealed that while women felt generally informed, they expressed a lack of choice in their care. Preoperative confusion, inadequate postpartum care communication, and delayed catheter removal were common concerns. The study emphasized the need for improved communication and shared decision-making to enhance satisfaction [20].

This study evaluated the comprehensive intervention effect of Manchester pain management and empowerment education model in postoperative rehabilitation by comparing the degree of pain, recovery indicators and nursing satisfaction after cesarean section between the observation group and the control group. The results show that this comprehensive intervention model is of great significance to improve postpartum recovery and maternal satisfaction.

In terms of pain management, VAS scores between the observation group and the control group showed statistically significant differences, and the observation group had better performance in postoperative pain management. Specifically, VAS scores in the observation group were significantly lower than those in the control group at 4 hours, 12 hours, and 24 hours post-operative resting and active states (*P* < 0.001). These results reflect the positive effect of comprehensive pain management programs, including interventions such as emotional support establishment, periodic goal setting, and individualized planning, on postoperative pain relief. At the same time, the program also helped women better express and evaluate their pain feelings, thus promoting the implementation of more appropriate pain management strategies.

In terms of postoperative recovery indexes, the first independent out of bed activity time and the first exhaust time in the observation group were significantly better than those in the control group (*P* < 0.001). This not only reflects the improved recovery of intestinal function after obstetrics and gynecology, but also the effect of early activity brought about by good pain control and enhanced rehabilitation education. These results emphasize the positive effect of comprehensive intervention after cesarean section on promoting physiological recovery. In addition, the average length of hospitalization of women in the observation group was shorter than that in the control group, and the success rate of first breastfeeding was higher than that in the control group, indicating that the overall recovery of women in the intervention group was more smooth, and the demand for hospital resources was less, which had positive significance from the perspective of economic burden and maternal satisfaction.

In terms of nursing satisfaction, the observation group scored significantly higher than the control group on all dimensions, including pain control method satisfaction, pain education satisfaction, and overall nursing satisfaction. This result was related to the maternal education on postpartum pain knowledge and skills, as well as close communication with the care team. This participatory approach and maternal-centered pain management strategies provide care that is more responsive to maternal needs and contributes to improved maternal subjective experience and satisfaction.

The limitation of this study is that the sample size is relatively small and it is a single-center study, which may be limited by the nature of variance and selection bias. Future studies can further verify the universality and robustness of the results of this study by expanding the sample size and adding multi-center participation. In addition, long-term follow-up of maternal recovery and changes in quality of life should be considered in study design to thoroughly evaluate the long-term effectiveness of integrated pain management.

## 5. Conclusion

In summary, the data of this study show that the comprehensive intervention combined with the Manchester pain management model and maternal empowerment education has a positive effect on pain control, physiological recovery and nursing satisfaction after cesarean section. These findings highlight the importance of implementing a holistic, maternal-centered pain management strategy in obstetric care practice.
